# Serum cytokine levels in patients with chronic low back pain due to herniated disc: analytical cross-sectional study

**DOI:** 10.1590/S1516-31802010000500003

**Published:** 2010-09-02

**Authors:** Durval Campos Kraychete, Rioko Kimiko Sakata, Adriana Machado Issy, Olívia Bacellar, Rogério Santos-Jesus, Edgar Marcelino Carvalho

**Affiliations:** 1 MD, PhD. Assistant professor, Universidade Federal da Bahia (UFBA), Bahia, Salvador, Brazil.; II MD, PhD. Associate professor, anesthetist and coordinator of the Pain Clinic, Department of Anesthesia, Universidade Federal de São Paulo (Unifesp), São Paulo, Brazil.; III PhD. Assistant professor and pharmacologist, Department of Anesthesia, Universidade Federal de São Paulo (Unifesp), São Paulo, Brazil.; IV PhD. Immunologist, Department of Immunology, Universidade Federal da Bahia (UFBA), Salvador, Brazil.; V MD. Psychiatrist and Statistician, Department of Medicine, Universidade Federal da Bahia (UFBA), Salvador, Brazil.; VI MD, PhD. Head, Department of Immunology, Universidade Federal da Bahia (UFBA), Bahia, Salvador, Brazil.

**Keywords:** Cytokines, Low back pain, Tumor necrosis factor-alpha, Interleukins, Interleukin-6, Citocinas, Dor lombar, Fator de necrose tumoral alfa, Interleucinas, Interleucina-6

## Abstract

**CONTEXT AND OBJECTIVE:**

The role of immune response and proinflammatory cytokines in the pathogenesis of chronic pain has been of growing interest. In order to evaluate whether there is any association between disc herniation and elevated cytokine levels, we measured cytokine levels in patients with chronic low back pain and in healthy subjects.

**DESIGN AND SETTING:**

Analytical cross-sectional study at the Pain Clinic of Universidade Federal da Bahia (UFBA).

**METHODS:**

Cytokine levels were measured using the enzyme-linked immunosorbent assay (ELISA) technique on 23 patients with low back pain (G1) and on 10 healthy subjects (G2).

**RESULTS:**

The levels of tumor necrosis factor-alpha [TNF-alpha] (G1 = 5.6 ± 2.3 pg/ml; G2 = 1.6 ± 0.5 pg/ml; P = 0.01) and interleukin-6 [IL-6] (G1 = 4.1 ± 3.0 pg/ml; G2 = 0.9 ± 0.4 pg/ml; P = 0.01) were higher in G1. There were no statistically significant differences in relation to interleukin-1 [IL-1] (G1 = 0.5 ± 0.3 pg/ml; G2 = 0.5 ± 0.1 pg/ml; P = 1) or soluble tumor necrosis factor receptor [sTNF-R] (G1 = 572 pg/ml ± 36; G2 = 581 ± 50 pg/ml; P = 0.87).

**CONCLUSION:**

The patients with chronic low back pain due to disc herniation presented higher levels of TNF-alpha and IL-6, but not of IL-1 or sTNF-R.

## RESUMO

**CONTEXTO E OBJETIVO:**

A função da resposta imunológica e das citocinas pró-inflamatórias na patogênese da dor crônica tem tido interesse crescente. Para avaliar se há correlação entre hérnia de disco e aumento de citocinas, foi medida a concentração de citocinas em pacientes com lombalgia crônica e em indivíduos sadios.

**TIPO DE ESTUDO E LOCAL:**

Estudo transversal analítico realizado na Clínica de Dor da Universidade Federal da Bahia (UFBA).

**MÉTODO:**

As concentrações de citocinas foram medidas pela técnica de ELISA (enzyme linked immunosorbent assay) em 23 pacientes com lombalgia (G1) e 10 sadios (G2).

**RESULTADOS:**

As concentrações de fator-alfa de necrose tumoral [TNF-alpha] (G1 = 5.6 ± 2.3 pg/ml; G2 = 1.6 ± 0.5 pg/ml; P = 0,01) e interleucina-6 [IL-6] (G1 = 4.1 ± 3.0 pg/ml; G2 = 0.9 ± 0.4 pg/ml; P = 0,01) foram maiores no G1. Não houve diferença estatisticamente significante para interleucina-1 [IL-1] (G1 = 0.5 ± 0.3 pg/ml; G2 = 0.5 ± 0.1 pg/ml; P = 1) e receptor solúvel do factor de necrose tumoral [sTNF-R] (G1 = 572 pg/ml ± 36; G2 = 581 ± 50 pg/ml; P = 0,87).

**CONCLUSÃO:**

Os pacientes com lombalgia crônica por hérnia de disco apresentam concentrações maiores de TNF-alpha e IL-6, mas não de IL-1 ou sTNF-R.

## INTRODUCTION

Low back pain is extremely prevalent. It impairs individuals’ quality of life and work capability, and thus has important social and economic implications.^1^ Approximately 60% to 80% of the United States population will experience back pain at some point during their lives and, at any given time, 55% suffer from low back pain associated with radicular syndromes. Moreover, about 1% of the United States population is chronically disabled because of back problems, and another 1% is temporarily disabled.^2-4^ Among a variety of etiologies for low back pain, herniated disc disease has been postulated as an important cause. It has been estimated that herniated disc disease could be present in 4% to 12% of patients with low back pain and could affect 5% of adults, according to population-based surveys.^5-9^ Sciatica symptoms are very persistent in nature over time, and up to one third of all such patients undergo lumbar surgery.^10^

Mechanical compression of peripheral nerve roots results in tissue damage, thereby causing inflammation with a direct effect on neurological function. Such injuries are potentially responsible for spontaneous discharges and increased amplitude of the electrical signaling response of the lumbar nerve roots, as demonstrated in animal models.^11^

It has been suggested that these injuries may modulate neuroimmune cascades, particularly the upregulation of cytokines in the damaged area, which may induce the expression of numerous algesic mediators that ultimately lead to pain.^12,13^ The extent of cytokine production is complex and may be influenced by the degree of nuclear exposure at the herniation site. Previous studies have examined whether circulating proinflammatory cytokine levels become elevated in syndromes associated with chronic pain, but mixed results have been reported.^14-17^ The cytokines that could present abnormal levels in blood and cerebrospinal fluid include interleukin-8 (IL-8), interleukin-1 (IL-1), tumor necrosis factor-alpha (TNF-alpha), interleukin-6 (IL-6) and soluble TNF receptor (sTNF-R).^18^ One challenge in interpreting the cytokine levels reported in many papers has been the limited information on healthy norms and reference values.^19,20^

## OBJECTIVE

The aim of this study was to evaluate the prevalence of elevated serum cytokine levels in patients with chronic pain due to herniated disc disease, compared with healthy subjects.

## METHODS

### Study population

After this analytical cross-sectional study had gained approval from the institutional ethics committee, patients were included following the signature of a written informed consent statement. In this manner, 23 consecutive patients with at least three months of back pain due to herniated disc disease were selected from the Pain Clinic of Universidade Federal da Bahia (UFBA). They were compared with 10 healthy subjects from the hospital community (with ages ranging from 20 to 65 years), without any previous history of back pain, who were used as controls.

The diagnosis was confirmed by means of magnetic resonance imaging (MRI) or computed tomography (CT) imaging of the spine, for all the patients. In addition, for patients to be included in the study, their pain severity had to be ≥ 5 points on a numerical rating scale (NRS), which ranged from zero (no pain) to 10 (worst imaginable pain).

The exclusion criteria were defined as the presence of one or more of the following: psychiatric disorders, systemic or inflammatory diseases, histories of allergy, presence of motor deficits, histories of blood dyscrasia, pregnancy, active infection, tumors, use of analgesic drugs during the preceding week, or inability to come to the hospital for evaluation.

All patients underwent standard history-taking and physical examination. Neurological findings (sensory and motor deficits and reflex dysfunction) and the straight leg-raising test were also evaluated by means of clinical examination. All the data were registered to facilitate statistical analysis.

In this study, the sample size calculation was based on different studies in the literature (between 10 and 30 patients) and on the fact that normal individuals do not present circulating proinflammatory serum cytokines. A difference in serum cytokine levels of at least 4.0 pg/ml between the healthy volunteers and the patients with low back pain was considered clinically significant. On the basis of other studies, we estimated the within-group standard deviation (SD) for serum cytokines as 3.5. For a power of 0.95 and alpha = 0.05, the sample size was about 20 patients.

### Laboratory determinations

Five milliliters of venous blood was drawn in the morning from the subjects and immediately centrifuged. The serum was stored at –20 °C. The serum levels of the proinflammatory cytokines IL-1 beta, TNF-alpha, IL-6 and sTNF-R were measured using a commercially available quantitative sandwich enzyme immunoassay technique (R&D Systems, Minneapolis, Minnesota, United States). Briefly, a microplate was coated with a monoclonal antibody that was specific for the cytokines, and standards and samples were pipetted into the wells. After washing, an enzyme-linked polyclonal antibody that was specific for the cytokines was added. The reaction was revealed by addition of the substrate solution.

### Data analysis

The variables did not present a normal distribution, and therefore nonparametric tests were used. The cytokine levels were compared between the study and control groups using the Mann-Whitney test. The Spearman coefficient was used to determine the relationship between cytokines and continuous variables. The chi-square or Fisher exact test was used when necessary, to test differences between proportions. The Statistical Package for the Social Sciences (SPSS) statistical software (version 10.0, SPSS Inc., Chicago, Illinois, United States) was used for data analysis, and statistical significance was determined as P values < 0.05.

## RESULTS

**Figure 1.**
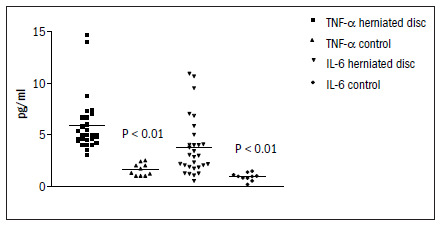
Distribution of tumor necrosis factor-alpha (TNF-α) and interleukin-6 (IL-6) in patients with herniated disc and controls.

Twenty-three patients were enrolled in the study: 52% were men and 74% were black. The mean age was 42.8 ± 7.0 years (median 42.0); the mean weight was 67.7 ± 9.0 kg (median 64.8); and the mean height was 165.1 ± 9.1 cm (median 167.0) (Table 1). The pain duration among the herniated disc patients was 81 ± 99 months (median 34.5) and the pain intensity as measured using the numerical rating scale was 9.0 ± 1.7 (median 10). The location of the herniated intervertebral disc was at the L4-L5 levels in 61% of the patients and at the L5-S1 levels in 39%. Pain was continuous in 78% of the subjects, with a daily frequency in 87%. The neurological findings were: a positive straight-leg-raise test (35%); hyporeflexia (17%); hypoesthesia (52%); and reduced muscle strength (4%) Table 2. As shown in Table 3, serum levels of TNF-alpha and IL-6 were statistically higher in G1 (P < 0.05). There were no differences in IL-1 beta or sTNF-R levels between the groups (P > 0.05), according to the Mann-Whitney test. The distribution of TNF-alpha and IL-6 levels in these two groups is depicted in Figure 1. The correlation coefficients between serum levels of TNF-alpha or IL-6 and pain intensity were, respectively, r_s_ = 0.28, P = 0.18; r_s_ = 0.32, P = 0.13; and in relation to duration of pain complaints were, respectively, r_s_ = 0.06, P = 0.78; r_s_ = 0.10, P = 0.64. There was also no correlation between the levels of proinflammatory cytokines and clinical parameters like age, weight and height (P > 0.05).

**Table 1.** Patients’ characteristics

	Gender	Age (years)	Weight (kg)	Height (cm)
G1 (n = 23)	12 (M); 11 (F)	42.8 ± 7.0	67.7 ± 9.0	165.1 ± 9.1
G2 (n = 10)	6 (M); 4 (F)	39.5 ± 4.5	65.3 ± 6.8	165.3 ± 6.7
P	0.7220*	0.1893†	0.4680‡	0.9502‡

G1 = herniated disc patients; G2 = healthy control subjects; P = statistical significance ≥ 0.05; M = male; F = female

* Fisher exact test

† Mann-Whitney test

‡ Student’s t test

**Table 2.** Neurological findings in the group of patients with herniated disc (G1; n = 23)

Positive straight-leg raise test	8 (35%)
Hyporeflexia	6 (17%)
Hypoesthesia	12 (52%)
Reduced muscle strength	5 (4%)

**Table 3.** Serum cytokine levels in herniated disc patients (G1) and controls (G2)

(pg/ml)	G1 (n = 23)	G2 (n = 10)	P
IL-1 beta	0.5 ± 0.3	0.5 ± 0.1	1
IL-6	4.1 ± 3.0	0.9 ± 0.4	0.01*
TNF-alpha	5.6 ± 2.3	1.6 ± 0.5	0.01*
sTNF-R	572 ± 36	581 ± 50	0.87

IL-1 beta = interleukin-1 beta; IL-6 = interleukin-6; TNF-alpha = tumor necrosis factor-alpha; sTNF-R = soluble tumor necrosis factor receptor

* P ≤ 0.05; n = number of patients; Mann-Whitney test.

## DISCUSSION

The present study demonstrates that individuals with herniated lumbar intervertebral disc disease have elevated serum levels of TNF-alpha and IL-6, compared with healthy subjects.

Disc herniation disease causes nerve root impingement, which leads to overexpression of cytokines and a complex network of biochemical reactions that can modify the transcription factors involved in gene expression, expand the glial cells in the general vicinity and thus cause neuronal hyperexcitability. The increased concentrations of these substances in the herniated disc tissue suggests that cytokines potentially have the ability to cause endoneural edema and nerve fiber demyelination.^21-23^ Furthermore, cytokines excite nociceptors, which suggests that they may play a critical role in peripheral hyperalgesia and pain behavior.^24,25^ Nonetheless, the potential involvement of these substances in disc herniation may be related to a local process. Thus, documentation of elevated serum levels of proinflammatory cytokines is an important finding and indicates that these molecules may be involved in systemic inflammatory reactions and hyperalgesia.

Although elevated serum IL-6 levels in individuals with an ongoing history of sciatic pain following discectomy have already been reported,^15^ no such elevation has been found in subjects with disc herniation and sciatica.^16^ However, proinflammatory cytokines show circadian rhythms and variations in peripheral blood, and the differences can potentially be related to the following factors: 1) the time of the day at which the blood samples were drawn, based on a study that demonstrated that IL-6 concentrations peaked in the evening;^15^ 2) IL-6 is a cytokine that increases in concentration in response to stressful conditions and may be affected by any emotional changes or symptom amplification;^26^ 3) cytokines may be released in a time-ordered sequence;^27^ 4) when an interleukin binds to its functional receptor, the complex is internalized;^28^ and 5) cytokines are also potent stimulators of the hypothalamic-pituitary-adrenal (HPA) axis, either singly or in synergy with other classes of cytokines, thereby causing glucocorticoid release.^29^ Thus, a dysfunctional HPA axis response occurring in some patients may result in elevated serum cytokine levels.

Nygaard et al. indicated that different types of disc herniation have different inflammatory properties.^30^ A recent study has demonstrated that intervertebral disc cells may produce TNF-alpha and IL-1 beta immediately after the onset of disc herniation.^31^

Koch et al. observed that increasing serum levels of proinflammatory cytokines (IL-1 beta, IL-2, IL-6, interferon-gamma [IFN-gamma] and TNF-alpha) correlated with increasing pain intensity in patients with chronic pain.^17^

High levels of proinflammatory cytokines have been reported in inflammatory and infectious diseases and can be correlated with disease severity.^32,33^ In this study, we did not find any other clinical illness that could explain the high levels of proinflammatory cytokines. On the other hand, our study population was small and, therefore, confounding factors could not be taken into account. Moreover, little is known regarding the impact of immune factors on pain from herniated discs. If proinflammatory circulating cytokines are mediators of pain and neuropathological changes in these sensory neurons, their inhibition constitutes an alternative to surgical treatment. This would decrease costs and postoperative complications. The opportunities for pharmacological interventions targeting the neuroinflammatory and neuroimmune components of various pathological conditions will be an exciting area of research. Thus, further research is needed to elucidate which of these processes are amenable to treatment and to determine the sensitivity and specificity of these observations for facilitating diagnoses, disease monitoring, and prognoses.^34^

## CONCLUSION

Despite the small number of subjects included in this study, the patients with chronic low back pain and disc herniation exhibited significantly higher levels of TNF-alpha and IL-6, but not of IL-1 or sTNF-R, compared with healthy subjects.
